# Realistic Dynamic Numerical Phantom for MRI of the Upper Vocal Tract

**DOI:** 10.3390/jimaging6090086

**Published:** 2020-08-27

**Authors:** Joe Martin, Matthieu Ruthven, Redha Boubertakh, Marc E. Miquel

**Affiliations:** 1 MR Physics, Guy’s and St Thomas’ NHS Foundation Trust, St Thomas’s Hospital, London SE1 7EH, UK; joe.martin@nhs.net; 2 Clinical Physics, Barts Health NHS Trust, St Bartholomew’s Hospital, London EC1A 7BE, UK; matthieuruthven@nhs.net; 3 Singapore Bioimaging Consortium (SBIC), Singapore 138667, Singapore; redha_boubertakh@sbic.a-star.edu.sg; 4 Centre for Advanced Cardiovascular Imaging, NIHR Barts Biomedical Research Centre (BRC), William Harvey Research Institute, Queen Mary University of London, London EC1M 6BQ, UK

**Keywords:** numerical simulations, phantoms, MRI, real-time, speech, upper vocal tract

## Abstract

Dynamic and real-time MRI (rtMRI) of human speech is an active field of research, with interest from both the linguistics and clinical communities. At present, different research groups are investigating a range of rtMRI acquisition and reconstruction approaches to visualise the speech organs. Similar to other moving organs, it is difficult to create a physical phantom of the speech organs to optimise these approaches; therefore, the optimisation requires extensive scanner access and imaging of volunteers. As previously demonstrated in cardiac imaging, realistic numerical phantoms can be useful tools for optimising rtMRI approaches and reduce reliance on scanner access and imaging volunteers. However, currently, no such speech rtMRI phantom exists. In this work, a numerical phantom for optimising speech rtMRI approaches was developed and tested on different reconstruction schemes. The novel phantom comprised a dynamic image series and corresponding k-space data of a single mid-sagittal slice with a temporal resolution of 30 frames per second (fps). The phantom was developed based on images of a volunteer acquired at a frame rate of 10 fps. The creation of the numerical phantom involved the following steps: image acquisition, image enhancement, segmentation, mask optimisation, through-time and spatial interpolation and finally the derived k-space phantom. The phantom was used to: (1) test different k-space sampling schemes (Cartesian, radial and spiral); (2) create lower frame rate acquisitions by simulating segmented k-space acquisitions; (3) simulate parallel imaging reconstructions (SENSE and GRAPPA). This demonstrated how such a numerical phantom could be used to optimise images and test multiple sampling strategies without extensive scanner access.

## 1. Introduction

### 1.1. Upper Vocal Tract and Dynamic Imaging Rationale

**Figure 1:**
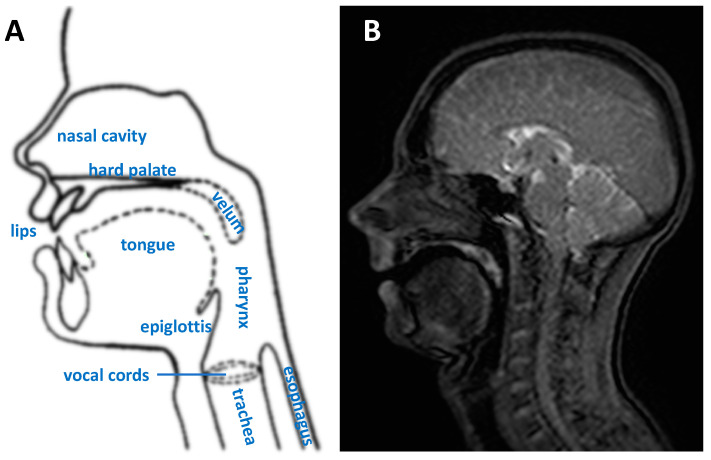
(**A**) A mid-sagittal diagram of the upper vocal tract highlighting the main speech organs or articulators. (**B**) A corresponding typical frame from a dynamic MRI scan during speech.

The upper vocal tract is an anatomical region covering the neck from the vocal cords to the mouth and nasal cavity (Figure 1). The production of speech is a complex process that involves numerous organs, referred to as articulators, including the lips, teeth and jaw, tongue, soft palate or velum, nasal cavity, the pharynx and vocal cords (or folds). Air from the lungs is forced through the vocal folds; their vibration produces a frequency and harmonics that can be controlled by the vocal cords. The other articulators form a network of connected resonant cavities that can be modified in shape and size allowing complex sounds to be formed [1,2]. At the back of the tongue lies the epiglottis, a flap that is open during respiration and speech and that closes to force food and fluids along the esophagus while preventing it from entering the trachea (Figure 1).

The ability to image dynamically the upper vocal tract using a non-invasive modality such as Magnetic Resonance Imaging (MRI) allows one to gain an understanding of the dynamic processes of speech and swallowing and this is consequently an active field of research [3,4]. The technique is particularly interesting in the field of linguistics to understand articulations of sounds by both native speakers (e.g., [5,6,7,8,9]) and learning in non-native learners (e.g., [10,11]) but also to study singing [12,13,14] and air-instrument music players [15,16].

Clinically, the functionality of speech organs can be affected by both inherited and acquired diseases [17] including cancers [18], vocal cords polyps [19], cleft lips and palates [20,21] and neurological conditions [22]. Dynamic and real-time MRI (rtMRI) of the upper vocal tract can provide an insight into the disease and help treatment planning. It has, for example, been used to study speech particularly in patients with repaired cleft palate and velopharyngeal insufficiency (e.g., [23,24,25,26]), while swallow studies have been used to study normal deglutition (e.g., [27,28]), including breastfeeding swallow [29] and a variety of conditions, particularly tongue reconstruction post-cancer [30,31,32,33].

### 1.2. Overview of Dynamic and rtMRI: Sequences and Acquisition Strategies

Over the years, multiple acquisition strategies have been used to achieve dynamic and real-time imaging of the upper vocal tract. At the onset, gated and triggered strategies were used. These techniques required the subject to repeat the speech task many times and any variation in its utterance could lead to synchronization issues with the acquisition [34,35,36]. Consequently, most recent approaches rely on the continuous acquisition of data during a single utterance of the speech sample [3,4]. Acquisitions are categorised as dynamic, near real time and real time depending on the reconstruction methodologies [37,38], although it is worth noting that some publications, especially older ones, are unfortunately referring to dynamic sequences as real time. For real-time acquisition, each consecutive frame is reconstructed and displayed on the scanner with very low latency, while the acquisition of the subsequent frame takes place. This type of acquisition should be preferred for the clinical assessment of speech where a speech and language therapist usually interacts with the patient during acquisition [25]. On the opposite end of the spectrum, dynamic implies that the raw data is reconstructed after the acquisition of the full speech sample has been completed. In this case, the reconstruction can occur on the scanner or completely off-line. The latter allows for more computer intensive reconstruction to be applied like iterative reconstructions (e.g., [39,40,41]) or for the user to use reconstructions methods where the frame rate can be adjusted post-acquisition, for example, using different sliding windows on the data (e.g., [42,43,44]).

Numerous image acquisition sequences have been used ranging from Turbo Spin Echo (TSE) (e.g., [45]) to steady state (e.g., [46,47]) and spoiled gradient echo sequences (e.g., [48,49,50,51]). The current recommendation is to use steady state sequences, balanced at 1.5T, with Cartesian acquisitions and spoiled sequences with non-Cartesian acquisitions [4]. It is worth highlighting that, although Echo Planar Imaging (EPI) methods are often used for fast acquisitions [52,53], their role has been so far extremely limited in upper vocal tract imaging with only a couple of publications using a hybrid-EPI sequence [51,54]. This is largely due to the presence of large air cavities making it hard to achieve a good image quality with EPI.

A wide range of k-space sampling schemes has also been used, including Cartesian [46,48,52], Cartesian with a spiral navigator [6,55], radial [41,56,57,58,59], and spiral [39,58,59,60]. In order to increase frame rates and achieve the desired temporal resolution for each type of investigation, undersampling and/or data sharing techniques need to be used. For Cartesian acquisitions, partial Fourier (e.g., [45,47]) is used as a first step but is usually insufficient on its own. As modern receiver coils are in fact a combination or array of coils, the next approach is to utilise that signal redundancy by using techniques known as parallel imaging [61]. They are so called because the signal of the multiple receiver coils is recorded concurrently, in parallel. It is consequently possible to reduce acquisition time by only filling a reduced proportion of k-space. The most common and commercially available parallel imaging reconstructions are SENSE [62] and GRAPPA [63]. SENSE reconstruction is performed in image space. First, low-resolution images are taken of the object to determine coil sensitivity maps, a distribution of the signal area visible to each coil. Aliased images are then acquired by reducing the number of phase encoding steps, and those images are un-folded by using the coil sensitivity maps and linear algebra. GRAPPA reconstruction is performed in k-space; the non-sampled portions of k-space are estimated using the surrounding k-space data. Those techniques are available commercially on MRI scanners and both SENSE (e.g., [39,47,51]) and GRAPPA (e.g., [13,64,65,66]) have successfully been applied to vocal tract imaging.

Finally, data sharing and sliding windows techniques can be employed to increase the temporal resolution (e.g., [42,43,44]); non-Cartesian acquisitions are particularly well suited to those methodologies.

### 1.3. Need for Optimisation and the Use of Phantoms

The complexity of the methodology used, combined with the inherent difficulties of imaging a moving organ necessitate extensive testing and optimisation of the imaging sequences to ensure that adequate spatial and temporal resolutions can be achieved with sufficient signal while artefacts are minimised to allow a correct diagnosis when developing a dynamic upper vocal tract protocol. Furthermore, speaking or swallowing in a supine position for long sessions, as is the case in speech MRI examinations, are by nature demanding on the subjects. Due to the extensive and expensive scanner time required, and despite advances made by research groups, the extensive optimisation remains a barrier and routine clinical imaging often remains at really low frame rates (1 to 3 fps [24,67]) or even static [68]. As a consequence, it would be advantageous for numerous groups to reduce the need or length of such sessions by carrying out some of the optimisation work on phantoms. However, moving MRI phantoms, although achievable, are notoriously difficult to manufacture [69,70,71]. An alternative is to develop a numerical phantom that can be used for initial optimisation. This approach has been successful in cardiac MRI with the MRXCAT phantom [72] and simulations based on acquired images [73]. Numerical phantoms have recently been created for dynamic liver imaging [74] and the entire abdomen [75]. Most phantoms are usually composed of organs or groups of organs with uniform signal. This approach allows to best visualise possible image artefacts, blurring and structure resolvability when optimising an imaging sequence while remaining anthropomorphic and retaining all physiologically important movement. Despite the proven benefits of numerical phantoms to develop and optimise image acquisition, this methodology has not yet been applied to the dynamic upper vocal tract MRI.

### 1.4. Aim of This Work

The aim of this work is to develop a framework to create the first numerical phantom for dynamic upper vocal tract imaging from previously acquired real-time speech images. This single slice phantom was then used to simulate images acquired at different frame rates and using different k-space trajectories (Cartesian and non-Cartesian). Finally, the phantom was used to simulate two parallel imaging acquisition methods, SENSE and GRAPA.

## 2. Materials and Methods

### 2.1. Numerical Phantom Development

**Figure 2:**
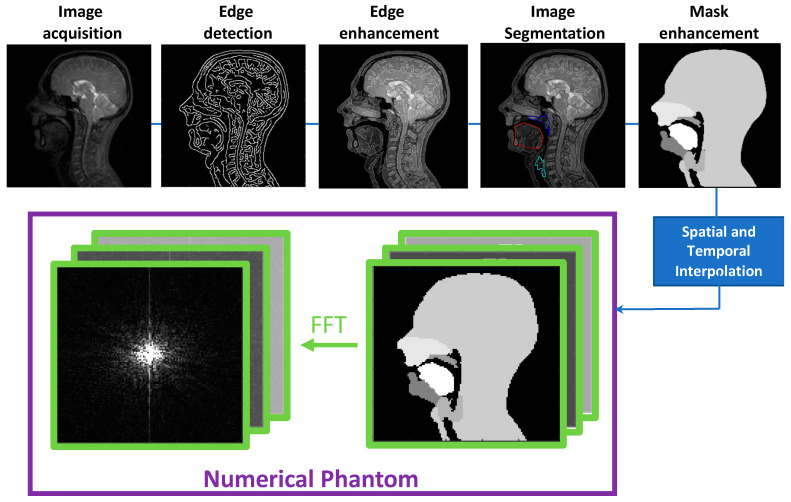
Software development framework used to develop the dynamic numerical phantom. The phantom is developed in image space from a real-time MRI video of a healthy volunteer acquired while speaking. The k-space numerical phantom is obtained by applying a fast Fourier transform (FFT) to the image domain numerical simulation.

A dynamic 2D numerical phantom was developed following a prototyping software development framework, which can be seen in Figure 2 [76]. The in-house software was implemented using MATLAB version 2016b (MathWorks, Natick, MA, USA) and is included in the Supplementary Material. In a similar way to other numerical phantoms, it is composed of a series of anatomical regions with a uniform signal picked to be similar to the contrast on the images but not dependent on the relaxation properties of the tissues. For this work, the following group of speech articulators were chosen: the velum, tongue, “epiglottis” (epiglottis + vocal cord area), “mandible” (lower jaw + lower lips and teeth) and “maxilla” (hard palate + upper incisor + upper teeth + nasal cavity). The remaining organs form a sixth region referred to as “head” in the rest of this article.

The phantom was created in image space. The dynamic MR images of speech used as the basis of the phantom were a mid-sagittal single-slice dynamic MR series of the upper respiratory tract, of a volunteer performing a standard speech sample designed to capture the full range of velocities and positions of the tongue and velum during speech for English speakers. The speech sample included: counting from 1 to 10; phonating nonsense (“za-na-za”, “zu-nu-zu”, “zi-ni-zi”); and saying “Bob is a baby boy”, “I saw Sam sitting on the bus” and “Tim is putting a hat on”. 

The images were acquired with ethics approval at St. Bartholomew’s Hospital, London, using a 3T Philips Achieva Tx MRI scanner in conjunction with a 16-channel head and neck coil (Philips Medical Systems, Best, The Netherlands). The protocol used is one of the sequences recommended by Scott et al. [47] and previously shown to adequately capture the motion of the velum and tongue [77]. The sequence details were as follows: a steady state free precession sequence, an echo time of 0.9 ms, a repetition time of 2 ms, a flip angle of 15°, a field of view (FOV) of 300 × 220 mm^2^, acquired pixel dimensions of 2.5 × 2.5 mm^2^, and a native frame rate 10 frames per second (fps). The dataset was chosen for its good image quality and included a total of 600 images.

Before segmentation, and to make this process easier and more accurate, the images were augmented with a “Canny” edge enhancement. A script was used to create and save a composite image of the original time series and added edges (normalised and then multiplied by 0.2 of the maximum intensity in the original image, this being found empirically to best aid segmentation).

**Figure 3:**
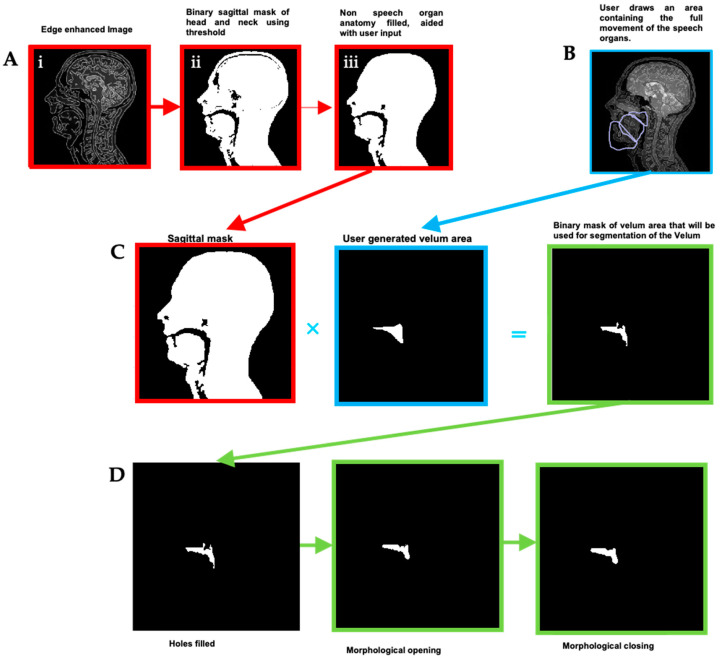
Creation and optimisation of the “head” and velum masks: (**A**) A binary mask of the head with the upper respiratory tract is made with some user input. (**B**) An area is drawn by the user that will enclose all potential positions the speech organ may move into through time. Overlap between masks of the different speech organs will be removed later through a combination of logical and morphological operators. (**C**) Initial segmentation: The binary mask of the user-generated velum area is multiplied on a pixel-by-pixel basis (Hadamard product) with the sagittal mask to create a binary image that is used to segment the velum. (**D**) Morphological operators are used to smooth the masks and fill holes.

A semi-automatic three-step process was then used to create dynamic segmentations for five relevant groups of speech articulators:

(1) Binary masks of the whole head with the vocal and speech organs visible were created using thresholding from the heads and some user input to ensure the upper respiratory tract remains distinct but that regions with zero value are filled in non-speech organs.
(2) Manually select a region containing each speech articulator. It must be sufficiently large to allow for a full range of movement of an organ of interest (such as the velum or tongue) and is outlined directly onto the image.
(3) Automatically segment and create a mask for each organ of interest at each time point, using the Hadamard product of the head mask and organ of interest mask at each time point, an example for the velum can be seen in Figure 3. This results in binary masks for each of the speech organs of interest for each frame in the original dynamic image set.

In order to create the final uniform organs, further automated binary morphological operations were applied in order to remove groups of isolated pixels, smooth rough protrusion and the edges of the mask and make sure that the segmented mask included all the organ, as part of the organs might be missing from the automatically segmented mask if artefacts (for example signal drop outs due to susceptibility) are present. This can occur more frequently in the tongue and velum, and the removal or isolated groups of pixels, and the filling of holes (principally in the tongue due to magnetic susceptibility artefact caused by signal drop out) as well as smoothing rough protrusions from the edge of the masks [78]. Finally, a structured series of logical operators are applied to the masks (such as Mask A AND NOT Mask B) to remove any overlap between them [78]. The segmentation, masks creation and optimisation steps for the “head” and “velum” regions are summarised in Figure 3.

The last step in the phantom creation was to interpolate the spatial and temporal resolutions. The temporal interpolation was carried out using the Euclidian distance transform and interpolating linearly between two given masks in the time series. A k-space version of the phantom can be created by fast Fourier transform (FFT). For this iteration of the phantom, the final resolution was set as follows: 30 fps, a simulated square FOV of 30 cm, an image matrix size of 256 × 256, and a spatial resolution of 1.719 × 1.719 mm^2^.

### 2.2. Numerical Phantom Testing

The phantom developed above was used to simulate fully sampled Cartesian and non-Cartesian trajectories, lower frame rate segmented Cartesian acquisitions and parallel imaging methods. For all the tests, relative image fidelity to the original fully sampled phantom was assessed in Matlab by calculating the root mean squared error (RMSE). The RMSE was calculated for each frame, and, when a mean is stipulated, it has been calculated over all the frames. When comparing acquisitions at different frame rates, only the temporal points common to all acquisitions were included in the analysis.

#### 2.2.1. Cartesian and Non-Cartesian k-Space Trajectories

In order to compare between Cartesian and non-Cartesian image reconstructions of the generated phantom dynamic time series, three different k-space sampling schemes were generated.

For the Cartesian images, a fully sampled k-space for each given time-point, *t*, was calculated using the FFT. A blipped EPI acquisition was also simulated, with a shift between odd and even k-space lines. For non-Cartesian sampling trajectories, spiral and radial trajectories that satisfy the Nyquist criterion were simulated using the non-uniform fast Fourier transform (NUFFT), respectively [79]. To create simulated images, the k-space trajectories, phantom images and density compensation function (based on Voronoi diagrams) [80] are used in the ‘NUFFT’ function from the ‘MRiLAB’ toolbox [81]. Noise-free as well as noisy images series (5% additive Gaussian noise) were generated.

#### 2.2.2. Simulating Lower Frame Rates

**Figure 4:**
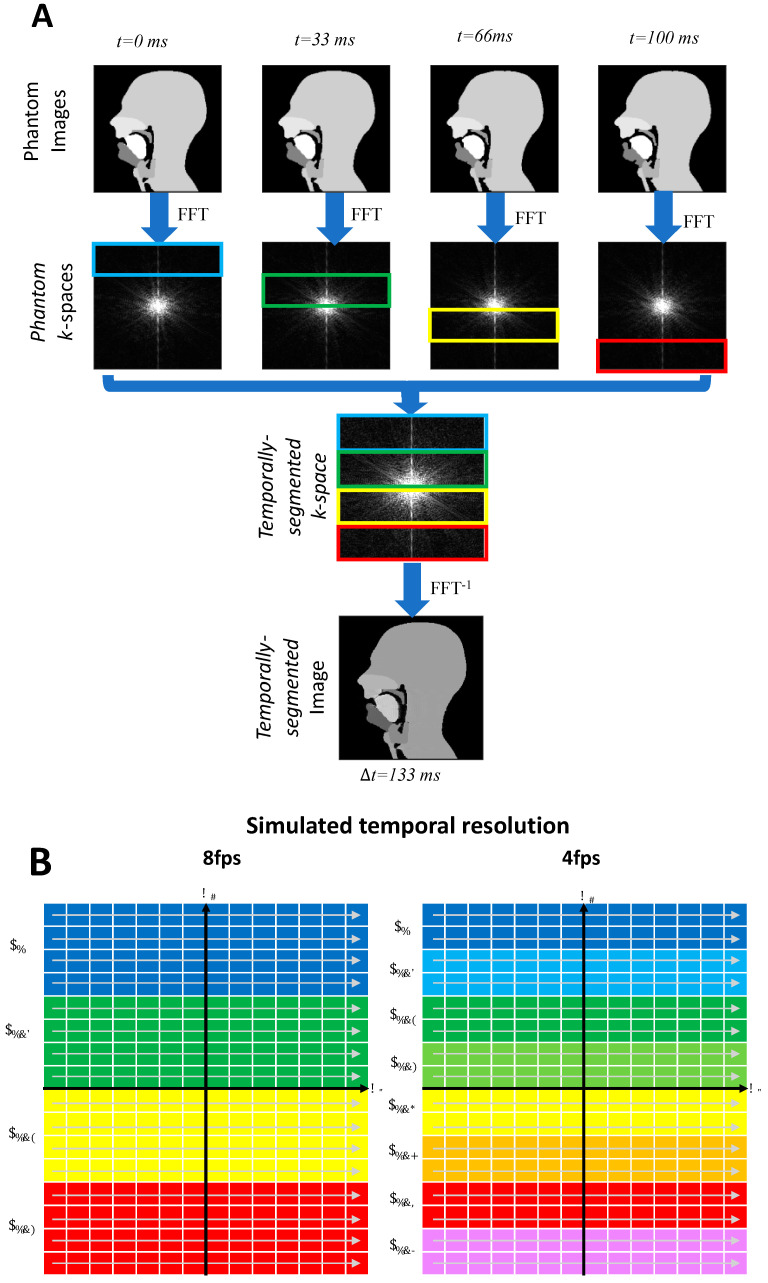
Segmented k-space Cartesian acquisitions can be simulated from the original 30 frames per second (fps) phantom. For example, a frame rate of 8 fps is obtained by assembling segments from 4 consecutive images from the original phantom (**A**). Likewise, a 4 fps will be created by assembling segments from 8 consecutive images from the fully sample numerical simulation (**B**). Segments are selected in k-space in a reverse linear fashion.

Lower temporal resolutions were created in two ways; one in the image space and one in the k-space. The former was carried out by averaging successive frames to reduce the frame rate. The latter is more representative of an actual acquisition process. Lower frame rate Cartesian acquisitions of 2, 4, 8, and 15 fps were simulated from the k-space numerical phantom using a segmented k-space methodology described in Figure 4. (Note that, for ease of notation, the frame rates are rounded to the nearest integer).

#### 2.2.3. Parallel Imaging Simulations

**Figure 5:**
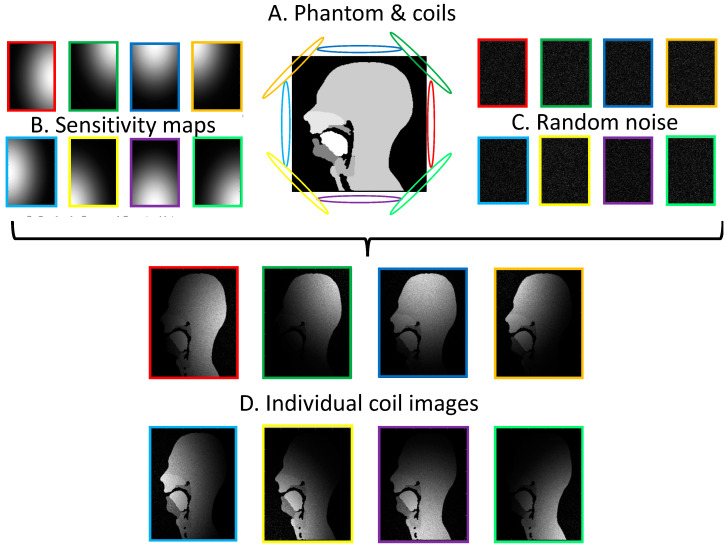
Creation of individual coil images for an 8-element array coil. The Hadamard product of the phantom images (**A**) with the coil sensitivity maps (**B**) and randomly generated Gaussian noise (**C**) results in simulated individual coil images (**D**).

Parallel imaging makes use of coil arrays to under-sample the acquisition [82]. The reconstruction of the under-sampled data relies on the signal from each coil and a priori knowledge of the signal distribution received by any coil in the array; this is known as a coil sensitivity map. Individual coil images can be created from the numerical phantom for any coil selection. The process for an 8-element coil is illustrated in Figure 5. Simulated coil arrays of 2, 4 and 8 elements were used in the experiments.

The GRAPPA [63] and SENSE [62] reconstructions were performed using the Berkeley Advanced Reconstruction Toolbox (BART) toolbox, an open-source image-reconstruction framework for computational MRI allowing efficient implementations of many calibration and reconstruction algorithms for parallel imaging and compressed sensing [83].

In order to investigate the effects of some of the phantom parameters on the quality of the reconstructed images, a number of simulated dynamic series parameters were investigated by varying the acceleration factor (R), the frame rate (fps), the number of fully sampled lines for the auto-calibration signal (ACS) and number of coils. Those were compared to a fully sampled simulated segmented acquisition at the same frame rate created in Section 2.2.2.

In addition to the RMSE analysis, the quality of the reconstructed images was assessed qualitatively by an experienced user (JM) using a binary scale: “Are the velum and tongue discernible?” (yes/no) and “Are aliased image repetitions/significant artefacts are apparent?” (yes/no).

## 3. Results and Discussion

### 3.1. Phantom Development

A framework was successfully implemented to develop the first numerical phantom for dynamic imaging of the upper vocal tract based on previously acquired rtMRI data. The framework essentially has three stages: (1) the creation of anatomical regions of interest throughout the original data, (2) interpolation to create the image space phantom and (3) Fourier transformation to create the k-space phantom.

The challenging and time-consuming stage is the creation of the anatomical regions. Those were obtained through a semi-automated segmentation process, as the manual segmentation of such a large number of images would have been extremely time consuming and automatic segmentation methods of the upper vocal tract are limited to delineating articulator surfaces [84,85,86,87].

**Figure 6:**
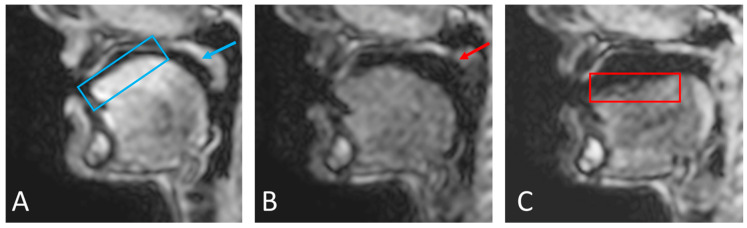
Varying image quality through the frames will have an effect on the initial automatic segmentation. While the velum (blue arrow) and tongue surface (blue box) can be easily automatically segmented in image (**A**), the automatic segmentation of the velum (red arrow) in image (**B**) and the tongue (red box) in image (**C**) will need manual corrections because of the signal drop-outs and artefacts caused by off-resonance.

First, an automatic segmentation was run based on edge detection and contrast between tissues. Although this gives a relatively good result, manual adjustments were required especially in regions where signal drop-out due to off-resonance and susceptibility are known to occur. In the upper vocal tract, this is particularly the case when the velum is in a high position and at the back of the tongue in certain phonics [4,8]. Example images are shown in Figure 6 to illustrate this problem in the velum and the tongue. However, all regions that were not fully anatomically correct were successfully corrected with the extra steps described in the methodology (Figure 3). It is worth noting that the subject used for this study did not have any dental work or orthodontics; those can impact the image quality [88] and would lead to increased problem when segmenting the images; hence, more corrections would be needed to create anatomically correct regions in the phantom.

Artificial-intelligence-based segmentation methods of the upper vocal tract have started to emerge and, in the future, could represent a suitable alternative for segmentation. However, they are only detecting the air–tissue interface [89,90,91], with some also detecting with which organ it is in contact with [92,93,94] and one fully segmenting the airway [95]. However, currently, none fully segment the articulators.

The next two stages are straight forward. First, various methodologies were pursued to find the best method of interpolating between the masks to create the increased temporal resolution. Optical flow pixel velocities were calculated but attempts to use these to create continuous deformation of the masks led to blurring and smearing of the image [96]. This smearing effect was again seen when attempting to perform non-rigid deformation using b-splines [97]. To avoid smearing of the masks, image interpolation between the masks was used, utilizing the Euclidian distance transform [98]. To avoid excessive interpolation, higher native frame rates could be used; however, image quality tends to worsen at higher frame rates, and this would make the segmentation in the first stage more challenging. The result of the interpolation is the image space phantom that just requires Fourier transformation to obtain the k-space phantom.

**Figure 7:**
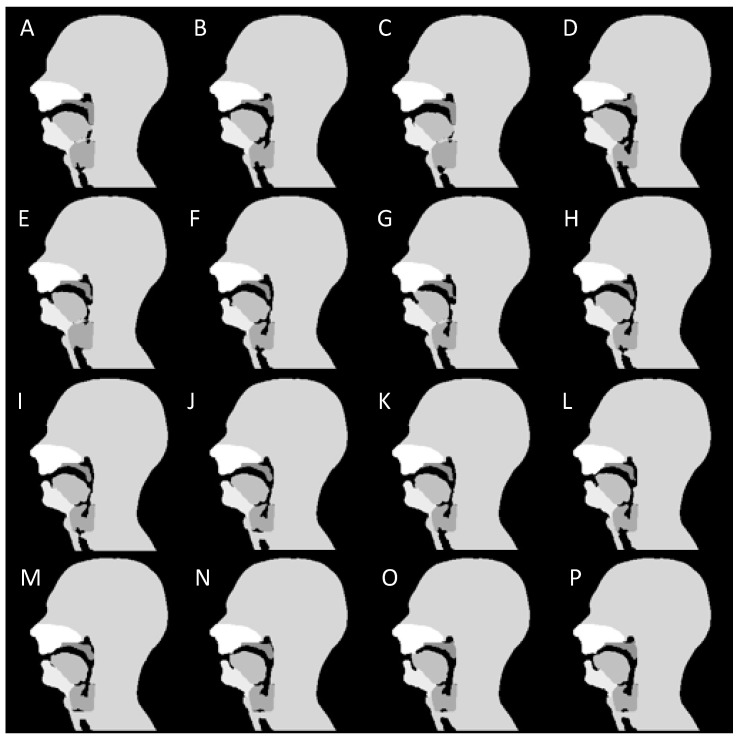
Sixteen successive frames (**A**–**P**) during the non-sense phonation “za-na-za”. The first 4 frames are during the end of the initial “za” and the soft palate is closed (**A**–**D**). The soft palate opens in the next frame at the beginning of the sound “na” (**E**) and recloses at the beginning of the next “za” (**M**) and remain closed thereafter **(N**–**P**). During the sound “na” (**E**–**L**), forward movement of the tongue can also be seen.

Following the three-stage process, a dynamic numerical phantom of the upper vocal tract during speech was successfully developed from real-time MR images acquired at 10 fps. The Matlab code for the numerical simulation (File S1) and a movie of the dynamic phantom (Video S1) are shown in the Supplemental Materials. Successive frames during the non-sense phonation “za-na-za” demonstrate velopharyngeal closure and opening are shown in Figure 7.

The phantom consists of six anatomical regions, five groups of speech articulators and one including non-speech organs, mainly the head and neck regions, each region with a homogeneous image intensity. This implementation of the phantom has a temporal resolution of 30 fps or 33 ms per image and a spatial resolution of 1.719 × 1.719 mm^2^. According to the recommendation article by Lingala et al. [4], this temporal resolution covers most speech imaging applications, including studying sustained sounds, velopharyngeal closures, velic motion, tongue movement, coarticulation events and consonant constructions, while the spatial resolution is sufficient for all. Only studying closures of the alveolar trill, a consonant sound not present in English, might require a slightly higher temporal resolution. Recent dynamic studies of deglutition have been carried out with frame ranging from 4 to 25 fps [27,99,100,101].

At present, the signal intensity in the different regions is not based on MR tissue properties. This is not a drawback, as most dynamic images are about analyzing timings and shapes of the different articulators and not achieving a particular tissue contrast. However, other static [102] and dynamic [72] numerical phantoms have successfully integrated tissue properties and this will be integrated in future development.

**Figure 8:**
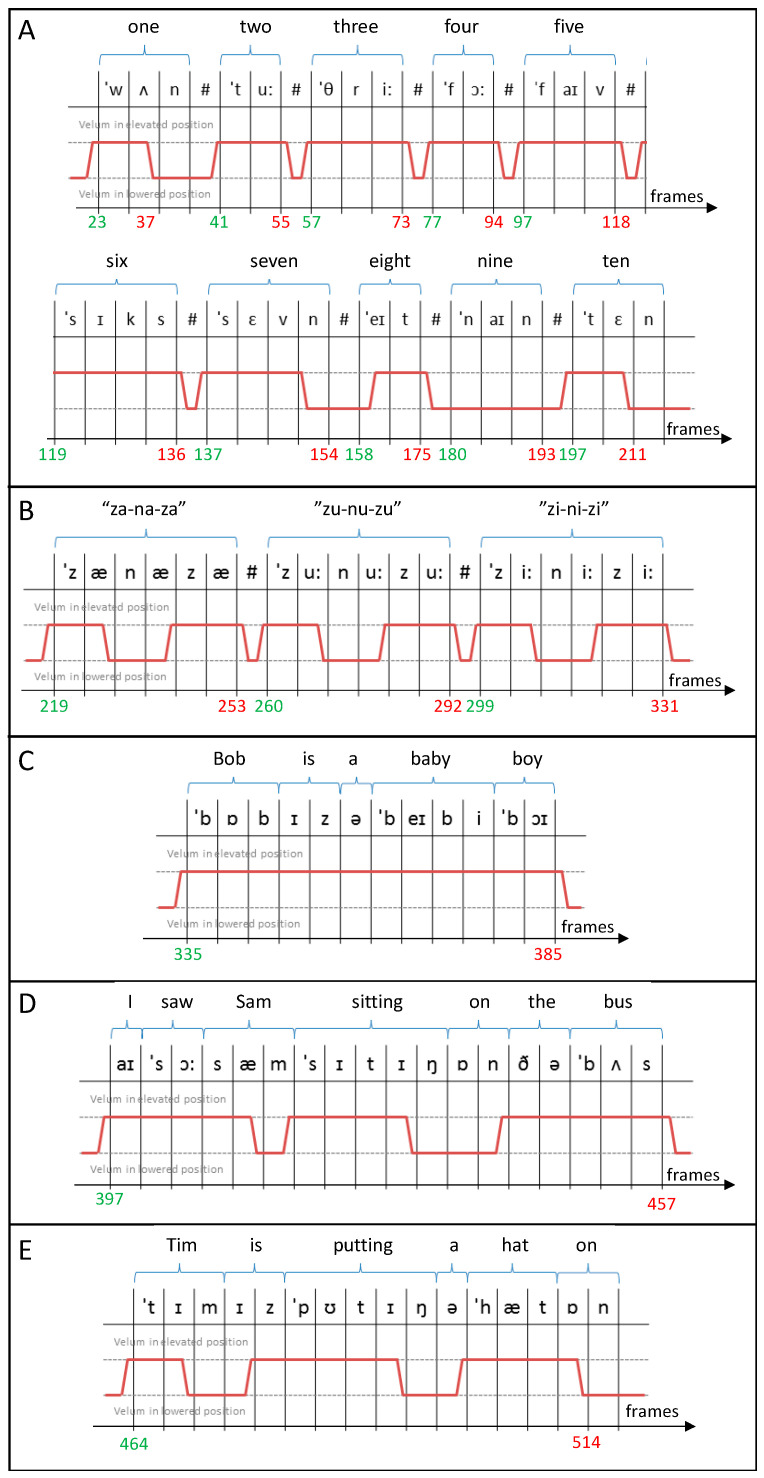
Details of the speech sample used in the numerical simulation including counting (**A**), non-sense phonation (**B**) and sentences (**C**–**E**). For each part of the speech sample, the phonetics sounds are included along with the position of the velum (red line) and the starting (green) and ending (red) frame number.

The phantom is based on a speech sample devised for clinical assessment of velopharyngeal insufficiency and a diagram of the sounds is given in Figure 8. The same process could easily be followed to created small numerical simulations for individual phonics.

### 3.2. Cartesian, Radial and Spiral Trajectories

**Figure 9:**
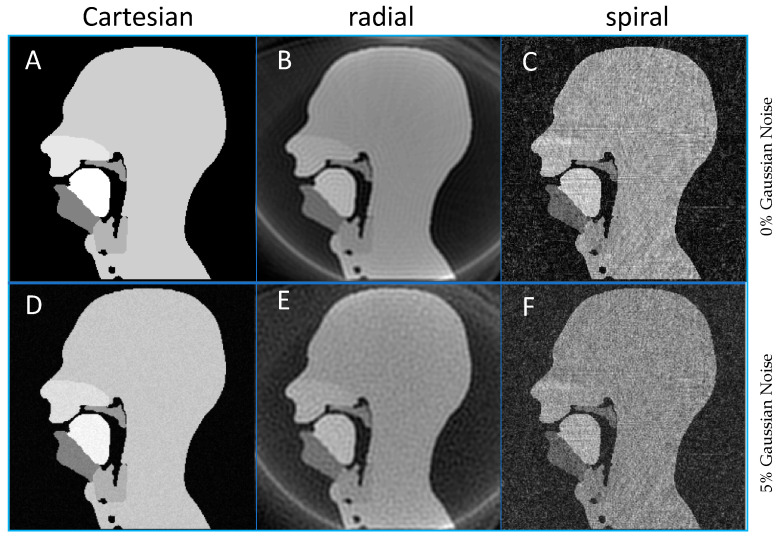
Example frames for a fully sampled k-space using different trajectories and noise level. (**A**,**D**) Cartesian, (**B**,**E**) radial, (**C**,**F**) spiral acquisitions with 0% (**A**–**C**) and 5% (**D**–**F**) noise added prior to Fourier transformation.

An example of reconstructed images for Cartesian, spiral and radial trajectories can be viewed in Figure 9, and the full movies of the three k-space trajectories without noise can be found in the Supplementary Materials, Videos S1 to S3. Both spiral and radial sampling trajectories allowed the individual speech organs to be viewed, which is the basic functional task required of these images in clinical speech MRI. The radial images showed the intrinsic ring aliasing artefact associated to it, as well as Gibbs artefacts near the edges of each of the speech organs, the latter of which has been reported in clinical radial imaging and is caused by the re-gridding process [103]. In the spiral images, a very streaked background noise was apparent across both of the images and this is again reported in clinical imaging as an effect of re-gridding [103] and, in this case, is an effect of multiple uncorrelated aliased images. Unlike in Cartesian imaging, correlated aliased repetitions observable in non-Cartesian acquisitions can obscure the anatomy of interest; these aliasing artefacts would not affect the diagnostic efficacy of the image.

In quantitative terms, the RMSE was greater for radial than spiral (22.1% to 18.6%) without added noise. However, the noise had little effect on the radial images RMSE, 22.6% corresponding to a 2.26% increase, whereas it led to a 32.80% greater RMSE for the spiral trajectory (24.7%). However, the identification of the speech organs is not impaired; in particular, the velum is clearly visible throughout all simulated images and all velopharyngeal closures (when the velum touches the back of the throat) are still clearly identifiable. Consequently, despite the noise and artefacts, these images are of equivalent diagnostic quality as the original Cartesian images.

**Figure 10:**
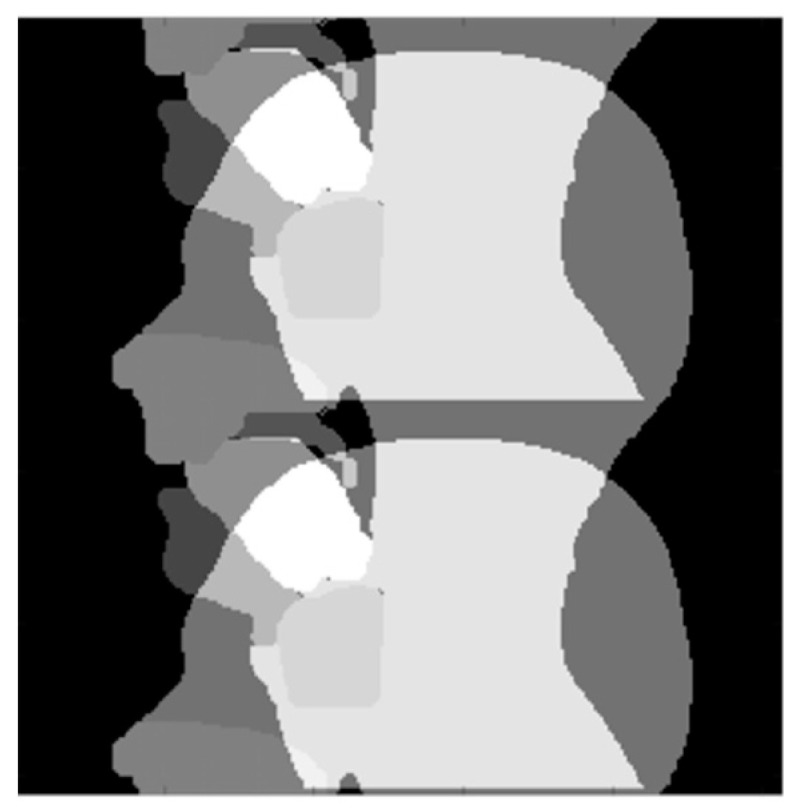
A frame from the blipped Echo Planar Imaging (EPI) simulation exhibiting Nyquist N/2 ghosting due to misalignments in the centres of odd and even k-space lines.

The blipped EPI simulation clearly demonstrated Nyquist N/2 ghosts (Figure 10) observed in the uncorrected images acquired with this method. Those are caused by differences in timing between odd and even k-space lines causing their centres to be misaligned [104,105].

The current numerical phantom does not include phase maps and, consequently, is not ideal for EPI simulation as those sequences are prone to distortion artefacts and require excellent shimming [106]. Those are particularly prevalent at air tissue boundaries, and this is why EPI is hardly ever used in the dynamic imaging of upper vocal tract [3].

### 3.3. Lower Frame Rates

**Figure 11:**
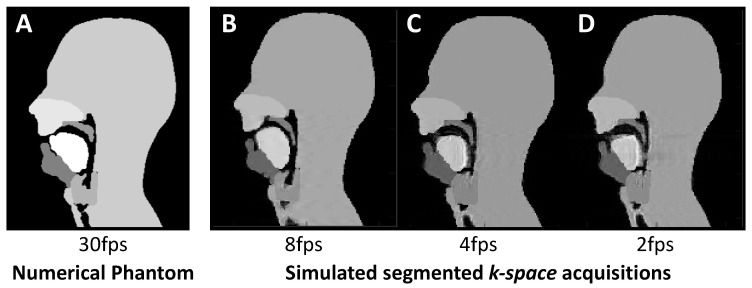
Example frames of the segmented k-space reconstruction at 8, 4 and 2 fps (**B**–**D**) exhibiting increasing temporal blurring when compared to the 30 fps numerical phantom (**A**).

Lower frame rates (15, 8, 4 and 2 fps) were created using two methods, one in image and one in k-space. The former can be used to create new simulations at lower frame rates by Fourier transforming the averaged image series; this method introduced lower level of temporal blurring and the mean RMSE values were 0.0279, 0.0383, 0.049 and 0.0739 for 15, 8 and 4 fps, respectively. The latter simulates a segmented k-space Cartesian acquisition at different frame rates. This introduces slightly more temporal blurring, and, in comparison, the mean RMSE values were 0.0593, 0.1185, 0.1289 and 0.1392 for 15, 8 and 4 fps, respectively. This is expected from a segmented k-space acquisition, which is known to suffer from temporal blurring; the lower the frame rate, the higher the number of segments needed per image and hence the higher the blurring [107]. Example images can be seen in Figure 11 and the movie for the 4 fps is available in the Supplementary Materials (Video S4). A lower frame rate clearly exhibits temporal blurring.

### 3.4. GRAPPA and SENSE Reconstructions

**Figure 12:**
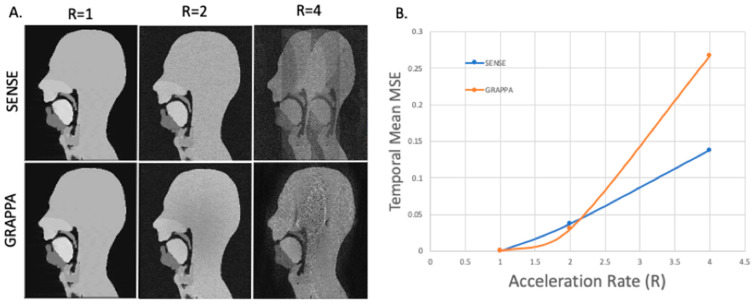
(**A**) GRAPPA and SENSE reconstructions for a simulated 8-element coil with increasing acceleration factor 2 and a 40-line auto-calibration signal (ACS) region (for GRAPPA). (**B**) Temporal mean root mean squared error (RMSE) compared to R = 1 for each of the associated reconstructions.

The full list of simulations and their corresponding parameters and image quality results are given in Table 1. For this experiment, the number of elements used varied from 2 to 8. Although this is lower than the number of coil elements in clinical head coils (typically 8 to 64); the phantom is only a single mid-sagittal slice and not all the elements of a commercial coil would contribute substantially to the signal received for this slice. Example images for a simulated 8-array coils and with and increasing acceleration factors along with the resulting mean temporal RMSE are shown in Figure 12. Videos of GRAPPA and SENSE reconstruction with an 8-element coil and an acceleration factor of 4 are available in the Supplementary Materials (Videos S3 and S4).

All the results and artefacts observed are in line with what can be expected from SENSE and GRAPPA reconstruction [62,63,82,108]. Increasing the acceleration rate hindered the ability of both the SENSE and GRAPPA reconstruction techniques to retrieve un-aliased images; as one would expect from theory and reconstructions, acceleration rates of 4 and above were undiagnostic. This is why acceleration factors used clinically are lower and typically between 1.5 and 3 [3,4]. The success of the SENSE and GRAPPA reconstructions differed depending on the parameters used. The results were not largely dependent on the temporal resolution despite an apparent simulated motion artefact. The quantitative and qualitative image results are similar for 2, 4, 8, and 15 fps, when considering just the reconstructions performed for all temporal resolutions. The temporal mean RMSE errors were fairly consistent, with only those at 15 fps being marginally worse. This may be due to its k-space only being comprised from two segments, and two phantom dynamic phantom k-spaces. The GRAPPA reconstructions were fairly dependent on the size of the ACS. At an acceleration rate of 2 and 10 ACS lines (of 256 PE lines), resultant images were consistent for all temporal resolutions: for 2 coils, the RMSE was poor (approximatively 14%) with significant aliasing artefacts apparent although the velum and tongue are discernible, whilst for 4 and 8 coils the RMSEs were satisfactory (4–7%) and the reconstructed images are diagnostically useful with the upper respiratory tract clearly visible, although some aliasing artefacts did remain outside the region of interest. The SENSE reconstructions for these same parameters were successful with no additional artefacts when compared to the fully sampled segmented k-space dynamic images. For an ACS region of 20 and 40 lines, artefacts were not apparent and the RMSEs are only marginally worse than those for SENSE.

## 4. Conclusions and Possible Directions for Future Work

We successfully devised a framework to create a dynamic numerical phantom of the upper vocal tract during speech. The phantom has a temporal resolution of 30 fps and can be used to simulate different acquisitions rates, k-space trajectories and reconstruction methods. The numerical phantom behaved as expected and was successfully used to simulate different temporal resolutions, from both image and k-space, test different Cartesian and non-Cartesian acquisition schemes as well as two parallel imaging techniques, SENSE and GRAPPA. These successful proofs of concept demonstrate that a numerical phantom could be used to reduce scanner time when developing and optimising new acquisitions for dynamic imaging of the vocal tract.

The iterative software development framework used to prototype the current numerical simulation allows for further improvement to maximise the phantom usefulness for both clinical and phonetics studies. In order to be able to cover all phonetics applications, including the study of alveolar thrills, the first step would be to increase the temporal resolution of the acquired segmented images up to 20 fps, which will allow the sampled k-space interpolated to be increased to 60 fps (16.7 ms temporal resolution). As dynamic images of the upper vocal tract are known to suffer from off-resonance artefacts, especially when the soft palate touches the posterior pharyngeal wall [4,8], a valuable addition would be to incorporate off-resonance maps to the numerical simulation. Furthermore, in this first phantom iteration, the signal intensity in the different regions is not based on MR tissue properties. Although not a drawback as most dynamic images are about analyzing timings and shapes of the different articulators and not achieving a particular tissue contrast, it would be an interesting addition for a closer simulation of certain pulse sequences. This has been achieved in other types of numerical phantoms [74,102] and would require the acquisition of T1 and T2 maps on the same subject as the dynamic MRI data.

The use of a different type of sequences could be investigated to see if the segmentation could be more automated; the use of spoiled gradient echo is currently prevalent at 3T, and hybrid EPI images could also be investigated as it has been previously used in cases where susceptibility artefacts were problematic [109].

As our primary interest is the clinical study of velopharyngeal closure, the speech sample used in this prototype was a typical clinical one that include a series of words and short sentences. However, the current software development framework can be easily used to create numerical simulations from dynamic MRI scans of individual phonics [110] or of swallow studies [27,28]. For the latter, the bolus would have to be segmented individually to create an extra region in the numerical simulation.

For a more advanced testing of future numerical simulations, more advanced parallel imaging techniques, such as radial and spiral GRAPPA and SENSE, would be another logical extension. Time parallel imaging techniques, where kernels are calculated not only from all coils but also across adjacent sampling times [111,112], would also be a valid extension to this model as they have started to be used in speech MRI [64,66].

## Figures and Tables

**Table 1:** SENSE and GRAPPA reconstructions of undersampled multi-coil images simulated from a dynamic speech phantom. Green shading indicates that the reconstructed images passed a given subjective fidelity criterion, whilst red shading indicates a failed subjective fidelity criterion.

Frame Rate (fps)	Number of Coils	Calibration Lines	R	Lines Sampled	MSE (%)		Velum & Tongue Discernible?		Aliasing Artefacts	
					GRAPPA	SENSE	GRAPPA	SENSE	GRAPPA	SENSE
2	2	10	2	128	14.01	4.09	Yes	Yes	Yes	No
	4	10	2	128	5.37	3.82	Yes	Yes	Yes	No
	8	10	2	128	4.13	2.46	Yes	Yes	Yes	No
	8	20	2	128	3.19	2.45	Yes	Yes	No	No
	8	40	2	128	2.91	2.46	Yes	Yes	No	No
4	2	10	2	128	14.00	4.27	Yes	Yes	Yes	No
	4	10	2	128	5.79	4.06	Yes	Yes	Yes	No
	8	10	2	128	4.65	2.62	Yes	Yes	Yes	No
	8	20	2	128	3.35	2.62	Yes	Yes	No	No
	8	40	2	128	3.062	2.62	Yes	Yes	No	No
8	2	10	2	128	14.16	4.35	Yes	Yes	Yes	No
	4	10	2	128	6.26	4.19	Yes	Yes	Yes	No
	8	10	2	128	4.82	2.72	Yes	Yes	Yes	No
	8	20	2	128	3.45	2.72	Yes	Yes	No	No
	8	40	2	128	3.17	2.73	Yes	Yes	No	No
15	2	10	2	128	15.10	4.56	Yes	Yes	Yes	No
	4	10	2	128	7.40	4.45	Yes	Yes	Yes	No
	8	10	2	128	6.59	3.01	Yes	Yes	Yes	No
	8	20	2	128	3.75	3.01	Yes	Yes	No	No
	8	40	2	128	3.46	3.01	Yes	Yes	No	No
	8	20	4	64	92.69	26.66	Yes	No	Yes	Yes
	8	40	4	64	13.78	26.66	Yes	Yes	Yes	Yes
	8	20	8	32	64.41	35.44	No	No	Yes	Yes
	8	40	8	32	>99	35.41	No	No	Yes	Yes

## Supplementary Materials

The following are available online at https://zenodo.org/record/3909619#.X0eWe0oRVPY. File S1: Matlab file of the numerical simulation. Please, cite this article when using it. Video S1: Numerical simulation (Cartesian, 30 fps). Video S2: Segmented k-space simulation at 4 fps. Video S3: A SENSE reconstruction with an acceleration of 4 for a simulated 8-element coil. Video S4: A GRAPPA reconstruction with an acceleration of 4 for a simulated 8-element coil.
